# Insects in Pet Food Industry—Hope or Threat?

**DOI:** 10.3390/ani12121515

**Published:** 2022-06-10

**Authors:** Jagoda Kępińska-Pacelik, Wioletta Biel

**Affiliations:** Department of Monogastric Animal Sciences, Division of Animal Nutrition and Food, West Pomeranian University of Technology in Szczecin, Klemensa Janickiego 29, 71-270 Szczecin, Poland; jagoda.kepinska-pacelik@zut.edu.pl

**Keywords:** companion animal nutrition, edible insects, pet food production, novel protein source, nutritive value, safety

## Abstract

**Simple Summary:**

Today, more and more is being said about issues related to resource depletion. This also applies to the raw materials necessary for food production. At the same time, the awareness of dog caregivers regarding their proper nutrition is increasing, and a balanced pet food should not only be nutritiously valuable but also in some cases hypoallergenic. These two considerations are the reason why food for dogs containing insects is becoming increasingly popular. Moreover, insects are nothing new in the diet of animals at all—they are a part of the diet in their natural environment.

**Abstract:**

Due to the increasing global population, the world cannot currently support the well-known techniques of food production due to their harmful effects on land use, water consumption, and greenhouse gas emissions. The key answer is a solution based on the use of edible insects. They have always been present in the diet of animals. They are characterized by a very good nutritional value (e.g., high protein content and contents of essential amino acids and fatty acids, including lauric acid), and products with them receive positive results in palatability tests. Despite the existing literature data on the benefits of the use of insects as a protein source, their acceptance by consumers and animal caregivers remains problematic. In spite of the many advantages of using insects in pet food, it is necessary to analyze the risk of adverse food reactions, including allergic reactions that may be caused by insect consumption. Other hazards relate to the contamination of insects. For example, they can be contaminated with anthropogenic factors during breeding, packaging, cooking, or feeding. These contaminants include the presence of bacteria, mold fungi, mycotoxins, and heavy metals. However, insects can be used in the pet food industry. This is supported by the evolutionary adaptation of their wild ancestors to the eating of insects in the natural environment. The chemical composition of insects also corresponds to the nutritional requirements of dogs. It should be borne in mind that diets containing insect and their effects on animals require careful analysis. The aim of this article is to discuss the nutritional value of insects and their possible applications in the nutrition of companion animals, especially dogs.

## 1. Introduction

By 2050, the world’s human population is predicted to be 9–11 billion [1,2]. Therefore, the priority is now food security and environmental issues related to the production and consumption of food. According to World Health Organization (WHO) estimates, up to 870 million people may suffer from malnutrition during this time due to lack of food [3,4,5]. This information is even more difficult to believe given the forecasts of shrinking arable land and increasing protein needs.

Land use is also an important issue. This term refers to the total amount of land needed to produce a given raw material, such as meat. However, the term covers not only the land needed for grazing in free range or in planted pastures but also for the area required for forage production. Currently, the demand for land resources and land use change are increasing, which will consequently lead to their exhaustion [6]. The livestock sector is the main user of land resources, accounting for about 30% of the world’s land area [7]. Ruminants (e.g., sheep, goats, and cattle) use land resources the most because they use both forage crops and graze natural or planted pastures. The deforestation caused by the expansion of pastures and fodder crops generated 8% of the total anthropogenic CO_2_ emissions [7]. As feed production is responsible for the major part of the impacts, insect production is also efficient in terms of land use compared with traditional animal production to the extent that it is more efficient in terms of feed conversion.

Livestock production is facing enormous sustainability challenges around the world, which are increasing in the European Union (EU) due to consumption problems related in particular to the health and environmental effects of eating meat as well as growing social concerns about animal welfare [8]. To address these challenges, the European Green Deal Action Plan has been developed. The European Commission has adopted a package of legislative proposals to adapt the EU’s climate, energy, transport, and tax policies to meet the 2030 net greenhouse gas reduction target of at least 55% compared to 1990 levels [9]. Moving to a sustainable food system can bring environmental, health, and social benefits as well as fairer economic benefits. The EU’s goals are to ensure food security in the face of climate change and biodiversity loss, reduce the environmental and climate footprints of the food system, strengthen the resilience of the EU food system, and steer the global transformation towards a competitive sustainable fork [9]. Despite the Farm to Fork strategy announced as part of the European Green Deal, assuming the pursuit of organic and sustainable farming and the promotion of a healthy diet based on plant foods, the EU budget allocated to the promotion of meat and dairy products is very high. Moving to a healthy diet by 2050 will require significant dietary changes. The global consumption of fruits, vegetables, nuts, and legumes will have to double, and the consumption of foods such as red meat and sugar will need to be reduced by more than 50% [10]. Despite this, currently, intensive and large-scale meat production dominates, which is associated with negative external environmental effects, such as the deforestation caused by the cultivation of soybeans and the emissions of gases harmful to the climate as well as competition for land between feed and food [11].

Currently, the main options for meeting protein needs are the use of protein from legumes, nuts, or seeds. In particular, the issue of soybeans used in animal nutrition is one of the topics discussed in the scientific context [12]. Ways to reduce and/or replace the soybean ration in livestock feed are the focus of many research projects in Europe [12]. Such projects are mainly aimed at reducing the import of soybeans from North and South America, which cause negative environmental effects through transport or deforestation [12]. In addition, Europe depends on soybean imports because soybeans are not endogenous in Europe and are only grown in small amounts. This also contributes to farmers and researchers looking for alternative sources of protein to at least partially replace soy in animal diets. In addition, most of the soybeans used as a protein in livestock production are genetically modified soybeans, which are not always accepted by consumers today [13,14]. There are countries in Europe that want to prohibit the use of genetically modified organisms (GMOs) in livestock nutrition altogether. An example is Poland, where this ban is planned to be introduced in 2024 [15].

In regions where both meat production and consumption increase with increasing incomes, emissions are projected to accelerate further. In the face of these challenges, it is necessary to reduce greenhouse gas emissions from all sectors of the economy, including livestock farming [16]. Therefore, the diversification of protein sources is inevitable, so it is recommended to lead to a partial replacement of animal proteins with alternative sustainable protein sources [17].

Insects are characterized by a rapid growth and development cycle, which greatly facilitates their production. Moreover, by-products of the agri-food industry can be used as a breeding substrate, and thanks to the high efficiency of feed processing, an average of 2 kg of fodder mass can be produced from 1 kg of insect biomass [18].

Body size is a key feature of organisms and is strongly related to the latitude and temperature in a given region [19,20].

Insect farming is characterized by lower greenhouse gas emissions than farming animals for slaughter [21]. Moreover, the benefits of breeding them can increase environmental and economic efficiency through dual production systems. Overall, the benefits of consuming edible insects influence the environment, the economy, and the health of society around the world [1]. For example, to meet the nutritional needs of a global population of 9 billion according to current dietary patterns, overall food production is projected to increase by 70% by 2050. This phenomenon will have serious consequences, including an additional 30% increase in global greenhouse gas emissions resulting from the breeding of animals for slaughter [16,22].

Given the environmental impacts of animal husbandry, including land use, greenhouse gas emissions, and water pollution, increasing production is not a sustainable solution to the protein needs to meet the nutritional needs of an ever-growing human population [23]. These solutions can be modified to provide benefits for the environment, economy, and society [24]. Currently, global organizations are calling for the search for alternative sources of protein, such as insects, and the conversion of breeding practices based on slaughtering animals into the breeding of edible insects [4].

Attention is also focused on less conventional sources, such as the use of microorganisms (algae, bacteria, or yeast) and the aforementioned insects. Overall, insects are an interesting sustainable source of protein. Their farming is characterized by low greenhouse gas emissions, low water consumption, and a smaller area of agricultural land used for their production. Moreover, it also has a high feed conversion efficiency [25].

A growing world population and the depletion of resources require a rethinking of eating patterns and habits, especially those related to meat consumption, which also applies to dog caregivers. This review characterizes the main insect species allowed in the European Union and the advantages and risks of their use in the food industry, in particular in the production of dog food.

As a consequence of the increase in the number of people, the number of companion animals also increases. According to statistics reported by the Fédération Cynologique Internationale (FCI), the approximate total number of dogs (purebred or not) in the world is around 147 million [26]. Dog nutrition is mainly based on dry food, and their industry is still growing; the annual growth rate of the pet food industry (average value over the last 3 years) is 2.6% [27]. More and more of this impressive number of pet caregivers are becoming more and more aware of their pets’ nutritional needs. The constant demand of caregivers for better quality products means that the pet food sector is becoming especially aware of providing nutritious food for the health and welfare of animals [28].

Therefore, the pet food industry is also looking for alternative sources of protein that could be substitutes for protein from poultry, cattle, and pigs. High hopes are placed on insects whose nutritional value is comparable to that of conventional protein sources.

Importantly, the term frequently used to refer to insects and their proteins is ”novel”, but the term is not entirely correct, as insects have always been the food of humans and animals. It should be emphasized that insects are also food in the natural environment of canids of the species short-eared wolf (*Atelocynus microtis*), big-eared fox (*Otocyon megalotis*), sand fox (*Vulpes rueppellii*), desert fennel (*Vulpes zerda*), gray wolf (*Canis lupus*), and Darwin’s quill (*Lycalopex culpaeus*) (Table 1). Insects can also account for up to 6% of the diet of wild cat species [29]. Therefore, the aim of this article is to discuss the nutritional value of insects and their possible applications in the nutrition of companion animals, especially dogs.

## 2. Nutritional Value

### 2.1. Protein and Amino Acids

When considering new sources of protein, it is important to determine the quality of the protein. Protein of animal origin is considered the benchmark in terms of nutritional value. Nutritional quality is defined as the ability of a protein to meet basic amino acid requirements and is usually based on the composition of the amino acid proteins and their digestibility. In recent years, new sources of protein have emerged that make it possible to shift to more sustainable food production.

Protein is the most expensive of all nutrients in both economic and ecological terms [34]. Protein provides essential amino acids and chemicals necessary in the processes of amino acid synthesis, gluconeogenesis, and energy transformation. 

Importantly, the protein content is usually calculated from the total nitrogen using the nitrogen to protein conversion factor (Kp) of 6.25. This factor overestimates the protein content due to the presence of non-protein nitrogen in insects. In a study [35], the specific Kp of 4.75 was calculated for the *Hermetia illucens*, and a Kp of 4.67 was calculated for *Tenebrio molitor*, while Homska et al. [36] obtained a Kp of 5.12 for *Hermetia illucens* larvae meal and a Kp of 4.91 for *Tenebrio molitor* larvae meal. When using insects as an alternative protein source, care should be taken to avoid overestimating the protein content due to the presence of non-protein nitrogen. To avoid overestimating the protein content of insects, the authors of these studies proposed to use the above-mentioned Kp [35,36]. What is more, the protein content of an ingredient should be calculated from the sum of individual amino acids [37,38]. 

Table 2 presents the contents of macronutrients and energy values in selected forms of insects.

The contents of nutrients in insects may vary depending on the species as well as the environment in which they live and what they were fed [43]. Insects are rich in protein with high nutritional quality In a study conducted by Udomsil et al. [44] the essential amino acids contents of crickets were comparable to those of egg, chicken, pork, and beef, which are considered the main protein sources of the dog diet.

Different diets, especially those with different protein contents, can affect the growth and nutritional value of crickets. In the studies by Bawa et al. [45], an assessment was made of the effect of commercial diets and other formulated diets on the nutritional composition and growth parameters of domestic crickets. It was shown that crickets can be effectively produced on a 22% protein diet to improve their nutritional value [45].

Nowadays, one of the most promising sources of protein is black soldier fly larvae (BSFL). The literature data indicate that BSFL consist, on average, of 40–44% crude protein [46]. In the research of Huang et al. [46], it was shown that the crude protein contents in BSF, *Tenebrio molitor* larvae, and cricket are 42.0%, 38.3%, and 32.6% DM, respectively. Table 3 shows a comparison of the contents of essential amino acids (EAA) in insects compared to conventional protein sources in feed and raw pet food materials.

The high lysine and threonine contents in both *A. domesticus* (4.49 and 2.30 g/100 g of protein, respectively) and *G. bimaculatus* (4.76 and 2.75 g/100 g of protein, respectively) [44] could help supplement cereal-based diets, which are generally low in these essential amino acids [54]. In most edible insects, lysine and tryptophan have been reported to be low. However, the low or limiting amino acids vary according to insect species and their diets [55]. The results of a study [44] demonstrated that both *A. domesticus* and *G. bimaculatus* could be used as dietary amino acid supplements that provide gratifying amounts of the essential amino acids for human health. In terms of EAA content, insects can be a valuable alternative to the fish meals and soybean meals commonly used in pet food production.

### 2.2. Fat and Fatty Acids

Insects are a very good source of fat with a high nutritional quality. The crude fat levels range from 14.41 to 37.1% and are usually higher in larvae than in imago, as is the case with mealworms. Mealworm larvae consist of 37.10% fat, while imago consist of 14.88% fat (Table 2). BSF contain about 35–40% lipids [46,56]. Interestingly, due to their high fat content, BSF larvae were used as biodiesel [57,58]. At the same time, BSF provided the highest amount of crude fat 36.2% DM. These results partially overlap with the information presented in Table 2. BSF can be considered a type of “energy” insect, rich in both proteins and lipids [46].

The fatty acid contents of insects may vary depending on the environment in which they live and the species as well as what they were fed [43]. The fatty acid profiles found in insects most likely reflect the fatty acid composition of the feed they were fed [59]. It has been shown that the fatty acid profile of crickets depends on their age [60]. Saturated fatty acids (SFA) are primarily a source of energy for the body. In insects, SFA levels range from 28.20 to 49.60% fatty acids and are comparable to the SFA contents of conventional protein sources, such as chicken breast (43.14% of fatty acids). The fatty acid content depends on what the insects are fed, e.g., in the studies by Ewald et al. [61], most of the BSFLs contained a high percentage of SFA (up to 76% of total fatty acids), which is higher than the value they obtained from Matin et al. [48].

Important acids are C10:0 and C12:0, which are easily digestible. Until now, the most common dietary source of C12:0 was coconut oil. The high content of lauric acid (C12:0) can be considered as a factor increasing the immune response of animals. This compound also shows antimicrobial activity against pathogenic bacteria such as *E. coli*, *Salmonella* sp., and *Clostridium perfingens* [62]. BSF larvae is particularly rich in C12:0 acid, in which it constitutes 28.6% of the total fatty acids. Lauric acid (C12:0) has been shown to account for approximately 40% of the total fatty acids in BSF larvae [63]. This value is much higher than what can be found in conventional protein sources, such as chicken (1.80% of fatty acids), beef (0.07% of fatty acids) and pork (0.13% of fatty acids). Research conducted, among other reasons, in order to determine the effect of nutrition on the fatty acid composition of abdominal fat in poultry showed that dietary BSFL oil significantly increased the amount of branched-chain fatty acids in broilers. The fatty acid composition of abdominal fat was influenced by dietary fat sources. In particular, chickens fed diets containing BSFL oil were characterized by a higher content of SFA, especially lauric acid, compared to chickens fed with corn oil [64].

The research of Jayanegara et al. [65] showed that mealworm oil and cricket oil were similar in terms of the main fatty acid profiles. These findings suggest that the oils from these insects are particularly rich in monounsaturated fatty acids (MUFA). Insects are especially rich in oleic acid (C18:1 n-9), and its best source is domestic cricket (44.60% of fatty acids), which contains twice as much of this acid as, e.g., pork (23.65% of fatty acids) (Table 4). An adequate supply of this acid is important because it determines the oxidative stability of a given fat.

There are two different groups of polyunsaturated fatty acids (PUFAs): the ‘n-3-fatty acids‘ and ‘n-6-fatty acids’. Both are considered as essential fatty acids (EFA) because they cannot be synthesized by monogastric animals/humans and therefore must be obtained from the diet or supplementation. They have the greatest impact on the nutritional value of fat, mainly due to linoleic acid (LA, C18:2 n-6) and α-linolenic acid (ALA, C18:3 n-3), which form the essential fatty acids (EFA). The proportions of polyunsaturated fatty acids (PUFA) are high in mealworm larvae (25.10% of fatty acids) and house crickets (29.50% of fatty acids).

It is worth remembering that an adequate supply of antioxidants, including vitamin E, is important in the diet of animals, the demand for which increases with the supply of PUFAs. The results of the studies by Cheseto et al. [66] found that insect oils are richer in fatty acids, flavonoids, and vitamin E than vegetable oils. The results also suggest that the presence of fatty acids and higher levels of flavonoids and vitamin E in insects in greater amounts than in vegetable oils could serve as potential suitable biomarkers for their nutritional value for use as food ingredients. Of the n-6 fatty acids, the concentrations of linoleic acid (LA) determined in all the oils in that study were higher than those found in conventional meat products such as fish, beef, and chicken [67].

All insect oils are generally low in n-3 fatty acids [65]. In the body, ALA, as a result of successive desaturation and elongation processes, is metabolized into long-chain polyunsaturated derivatives of eicosapentaenoic acid (EPA; C20:5 n-3) and docosahexaenoic acid (DHA; C22:6 n-3). They play an important role in preventing or alleviating lesions, including inflammatory diseases. The main source of the EPA and DHA in the diet are algae and fish that feed on algae. Among insects, BSF larvae are the most significant source of EPA (1.70% of fatty acids) and DHA (0.70% of fatty acids).

Interestingly, research by Seo et al. [68] shows that food with BSFL can be successfully used in the nutrition of older dogs. Moreover, feeding with BSFL for 12 weeks lowered the serum cholesterol levels of the dogs at the end of the experiment.

As shown in Table 4, insects have comparable fatty acid contents to conventional animal sources, which proves that they can also be a good alternative in terms of their fatty acid content.

**Table 4:** Fatty acid composition (% of total fatty acids) in selected insect species and conventional protein sources.

Fatty Acids	Mealworm Larvae [69]	House Cricket Imago [44]	Black Soldier Fly Larvae [61]	Chicken (Breast) [70]	Beef (Intercostal Muscle) [71]	Pork (*Longissimus dorsi muscle*) [49]
C10:0	0.02	0.03	<0.5	0.73	0.05	0.16
C12:0	0.37	0.18	28.6	1.80	0.07	0.13
C14:0	3.13	0.86	6.1	3.62	3.15	1.67
C16:0	19.50	31.20	12.6	23.99	30.39	26.22
C18:1, n-9	44.60	25.80	25.10	31.81	41.02	23.65
C18:2, n-6	24.00	27.90	12.50	16.62	2.51	23.43
C18:3, n-3	0.91	1.39	3.40	0.89	0.23	0.45
C20:5, n-3	0.13	0.12	1.70	0.17	0.05	0.21
C22:6, n-3	0.07	0.00	0.70	0.00	0.05	0.44
SFA	28.20	42.30	49.60	43.14	49.92	39.95
UFA	71.60	56.30	50.50	57.16	50.08	60.04
MUFA	46.50	26.80	31.80	36.96	46.10	24.49
PUFA	25.10	29.50	18.70	20.27	3.98	35.55

### 2.3. Minerals

Insects, apart from being rich in protein and fat, are also an important source of minerals. The insects’ internal soft tissues are covered with a hard protective layer known as the exoskeleton. It performs several functions in the bodies of insects, acts as a protective shell, and is designed to facilitate metamorphosis. The exoskeleton is rich in chitin and is excreted from the body during metamorphosis. Insects have a protein-rich exoskeleton rather than a calcified skeleton, so the mineral content of most insects is relatively low [72]. While the availability of phosphorus in plant sources is low, nearly 100% of phosphorus is found in insects. Mostly, insects are good sources of trace elements, including iron, zinc, copper, manganese, and selenium. Finke [72] found, however, that the mineral composition is mainly a reflection of the material that insects were fed. Common animal protein sources have an excess of phosphorus over calcium.

While the exoskeleton of most insects is primarily composed of protein and chitin, black solder fly larvae [73] have a calcified exoskeleton in which calcium and other minerals are incorporated into the cuticle. Therefore, they can contain high levels of calcium [74]. As reported by Liu et al. [75] the content of calcium reaches up to 2900 mg/100 g in mature larvae, while the content of calcium in the DM can be significantly higher in the E-prepupa stage (3000 mg/100 g) than in the mature larval stage. In the same studies, the phosphorus content in the E-prepupa stage (620 mg/100 g) almost doubled that in mature larvae (350 mg/100 g), and the calcium-to-phosphorus ratio was 4.84–8.28:1, respectively.

As reported by Udomsil et al. [44], in cricket the calcium content can reach up to 149 mg/100 g, while the phosphorus content can reach up to 899 mg/100 g. The mineral composition in general probably largely reflects the food sources for insects, both those that are present in the gastrointestinal tract and those that are incorporated into the insect’s body as a result of the food it consumed. For example, the calcium contents of wax worms, house crickets, mealworms, and silkworms can all be increased 5- to 20-fold when fed a high-calcium diet. This increase in calcium appears to be solely due to the residual food in the gastrointestinal tract, with little of the calcium being incorporated into the insect’s body [76].

Table 5 shows mineral composition of selected insect species in comparison with common livestock species.

Insects are a good source of Ca and P, which are important in the growth of young animals, and as shown in Table 5, the meat species have an unfavorable Ca to P ratio, i.e., an excess of P in relation to Ca. Excess P is a factor limiting Ca absorption, which leads to kidney disease. The meat diet for dogs has a precise tendency to risk this nutritional imbalance.

### 2.4. Chitin 

Chitin is contained in the procuticle, the innermost layer of the epidermis, which in turn is the outermost layer of the insect exoskeleton [84].

The results of research by Henriques et al. [85] show that the chitin content in insects can vary largely in a range of 8–4600 micrograms of chitin per insect, depending on species, sex, and instar. The chitin content increases with the development of the larvae into further stages [86].

Chitin in insects is a problem due to its indigestion in humans and animals due to the lack of chitinase. Therefore, it is recommended to at least partially remove the chitin present in insects in order to increase their nutritional value. However, the removed chitin can provide an advantage when used in a low concentration as a feed additive, as the substance has antimicrobial properties against a wide range of microbial species. Its biological activity can be further enhanced by converting it into chitosan in a deacetylation procedure [87]. About half of the crude fiber analyte may be chitin, but the N-acetylglucosamine polymer contributes little to the crude protein value.

Future research should investigate whether this problem can be solved by consuming insects along with fruits such as *Bromeliaceae* and *Caricaceae*, which contain enzymes with chitinase-like activity [88].

### 2.5. Health Properties

In addition, insects are a source of antimicrobial peptides (AMPs) and lauric acid, which may be factors that improve the immune response and have a positive effect on the breakdown of the digestive tract microbiome [89,90,91,92].

Protein derivatives from BSF larvae (proteins and protein hydrolysates) contain a significant amount of low-molecular-weight peptides with antioxidant potential. The Mouithys-Mickalad et al. [93] study investigated the in vitro antioxidant potential of commercial BSF proteins and protein hydrolysates for radical scavenging activity, the modulation of myeloperoxidase activity, and the modulation of the neutrophil response. It has been found that BSF protein derivatives can effectively protect against the cell damage resulting from neutrophil and myeloperoxidase activity. The results of this study indicate that BSF protein derivatives can potentially be included in pet food formulas as health-promoting ingredients [93]. Edible insects and invertebrates are a potential source of antioxidant components, the effectiveness of which depends on their taxonomy and eating habits. More evidence is needed to understand whether the practice of eating insects and invertebrates may contribute to modulating oxidative stress in humans. The results show that the water-soluble extracts of grasshoppers, silkworms, and crickets show high antioxidant capacity (Trolox equivalent antioxidant capacity, TEAC) values, five times higher than fresh orange juice [94].

There is a low probability of a dog’s allergy to insect protein in food because insect protein is not very common in the nutrition of dogs. Insects, even when they are present in pet food, still have a small percentage due to the high protein content. Therefore, insect-based foods are increasingly used in the nutrition of dogs suffering from food allergies to conventional protein sources, such as poultry or beef. In case of dogs with food allergies, it is necessary to provide adequate food to minimize clinical symptoms. A risk of commercial pet food is possible contamination with foreign protein. Therefore, food containing hydrolyzed protein is not always suitable. For example, some dogs with a food intolerance to chicken protein will still react to hydrolyzed chicken liver, depending on the strength and type of the immune response. Therefore, for dogs with food allergies, it is worth introducing insect pet food into their diet [95].

However, with insects being used more extensively in the future as a protein source in companion animal food, further research into their allergenic and cross-reaction potential is required [96].

## 3. Digestibility

The feces of dogs fed a diet containing *Hermetia illucens* larvae had an increased concentration of chitin [97]. For extruded products with partially skimmed BSFL or YMW, each accounting for approximately 30% of the total dietary protein, the apparent digestibility of crude protein was 83.9 and 83.6% of the consumption in dogs. When dogs were given a mixture with YMW as an almost sole source of protein, the caregivers did not notice any changes in stool consistency [98]. For dogs fed commercial dry food based on lamb meal or BSFL, stool scores were not statistically different [99]. A study by Abd El-Wahab et al. [100] compared the incorporation of BSFL meal compared to poultry meal (PM) in the diet of dogs in terms of digestibility and fecal characteristics. Following this trend, PM or BSFL meal was added to replace approximately 30% of the dry weight of the basic extruded diet. Dogs fed the BSFL-based diet had a higher apparent protein digestibility (82.3%) compared to dogs fed the PM-based diet (80.5%). The apparent fat digestibility was higher in the groups fed the BSFL diet (94.5%) compared to those fed the PM-based diet (91.6%). The fecal consistency results for dogs fed both diets were within the acceptable range (well-formed and firm). The fecal dry matter content was higher in dogs fed the PM diet (33.0%) compared to those fed the BSFL diet (28.0%). These studies have shown that the inclusion of BSFL meal in dog food can be an appropriate source of protein without adversely affecting nutrient digestibility and stool quality [100]. In the studies by Penazzi et al. [101], two diets containing venison meal (CTRL diet) or black soldier larvae (BSF diet) as the main sources of protein were analyzed. Both diets showed similar nutrient digestibility values for dry matter, organic matter, ether extract, ash, and phosphorus. However, a statistical trend was observed indicating greater protein digestibility in the BSF diet compared to the CTRL diet. The calcium digestibility was higher in the BSF diet compared to the CTRL diet. An analysis of the digestibility of dog food containing insect meal as the sole protein source showed promising results in terms of showing similar values to the meat-based diet, indicating its suitability as a stable protein source in pet foods [102].

In the study of Freel et al. [103], it was found that BSFL meal and BSFL oil are well-tolerated by dogs, and their consumption does not have any physiological effects that would be of concern. Based on these data, BSFL meal and oil did not affect overall health, so they can be safely included in the dogs’ diet.

Studies [95] have shown that the use of domestic cricket or mulberry silkworm pupae can successfully replace poultry meal in the diet of dogs while not showing a negative effect on hematology or blood chemistry. As shown by Klinger et al. [95], insects may be a new source of food for patients with adverse food reactions. The aim of their study was to evaluate the effect of a new commercially available insect (mealworm larvae) protein-based diet on clinical signs in these dogs using the Canine Atopic Dermatitis Index (CADLI), Visual Analog Pruritus Scale (PVAS), and coat quality. A total of 20 dogs with atopic dermatitis due to a previously diagnosed adverse food reaction were enrolled in the study. This food was the only food given to the patients for 2 weeks. The lesion score improved in 12 of the 20 dogs. Only two dogs out of the 15 who completed the study showed a mild worsening of lesions (mean of 1.5 CADLI points). One dog’s skin lesion remained unchanged. The pruritus could be reduced in eight patients but remained unchanged in four dogs. Two more patients worsened slightly and one dramatically worsened. The coat quality was assessed in only 14 dogs. Six out of fourteen dogs showed an improvement in coat quality. The improvement in the assessment of hair lesions and the quality of the hair was significant, but there was no significant change in the assessment of pruritus. Based on these results, it can be concluded that the studied diet based on insect protein might be an interesting alternative for dogs with food intolerance [95].

## 4. Palatability Tests

Food for companion animals should not only be properly balanced but also tasty. Although the topic of the use of insects in pet food is gaining popularity, there is little reliable research on animal palatability tests. The purpose of these tests is to verify the animals’ nutritional preferences and determine which of the feeds are more preferred by the animal [104]. At the same time, this kind of research is crucial to understanding dog behavior and learning more about how to formulate a diet for dogs. It is well-known that different breeds of dogs have different dietary preferences, e.g., bassets and German shepherd dogs prefer fishmeal to saluki, which mainly choose corn flakes. The studies by Kierończyk et al. [105] analyzed the preferences of a wide population of different breeds of dogs in order to generally verify the suitability of insect attractants. As shown in this study, insects can be as effective in influencing dogs as commercial fodder containing flavor, while providing an additional source of high-quality crude protein and fat [105]. According to research [106], the inclusion of 8, 16, or 24% banded cricket meal in extruded food had no effect on food consumption by dogs [106].

Interestingly, Kowalska et al. [107] showed that a mono-diet based on insect meal can be successfully used in the nutrition of guppy fry. In these studies, the authors assessed the usefulness of insects in the diet of guppies (*Poecilia reticulata*). The fish had a choice of four attractants: fishmeal, black soldier fly meal, Madagascar cockroach meal, and superworm meal. The conducted research showed that superworm meal was the most preferred attractant by guppies [107]. Other studies assessing the effects of using full-fat BSFL meal as a substitute for fish meal and fish oil in the diet of Siberian sturgeon have shown that it is possible to produce extruded feed with a BSFL content of up to 30% and physical parameters suitable for feeding fish. In addition, an increase in feed acceptance was observed in facilities containing more than 10% and higher BSFL contents. In the groups where the feed contained 5 to 30% BSFL in the diet, the growth of the experimental fish and their feed conversion parameters improved [108].

## 5. Acceptance by Consumers

Despite existing literature data on the benefits of selecting insects as a protein source, their acceptance by consumers and animal caregivers remains problematic [109].

Consumer acceptance is a major obstacle to the successful commercialization of insects in western countries. The research by Naranjo-Guevara [110] investigated the factors that influence consumer acceptance. Acceptance among interviewees seems to be influenced by issues similar to those in other European countries, such as visual aspects and the awareness of the benefits. The influence of information on the desire to eat insects is an important result of this study. The data obtained confirm the need to inform and educate consumers about the environmental and health benefits of entomophagy. Successful efforts to implement entomophagy can increase insect food knowledge and inform (or educate) consumers about its benefits [110].

Sungmun et al. [109] conducted a survey of pet caregivers who were aware of and had an intention to purchase insect pet food. The results showed that 89.5% of the survey participants owned pets such as dogs, cats, etc., of which 55.6% knew about this type of pet food. Nearly half (48.5%) of the respondents intended to buy insect-based pet food, while the rest (51.5%) did not. The two main reasons why the respondents decided to buy it were the excellent nutritional value of insects and low allergenicity. The most important reason for refusing the purchase was a strong aversion to insects as food.

Trends in the use of insects in animal feed show that the market for edible insects as animal feed could reach USD 2.386 million by the end of 2029. Some companies are pioneers and have already started processing insects in compound feed and pet food [111]. On the European market, insect-containing food is becoming more and more popular in the nutrition of dogs, especially in those with food allergies. The most commonly used species is BSFL.

## 6. Hazards

Despite the many advantages of using insects in pet food, it is necessary to analyze the risk of adverse food reactions (AFR), including allergic reactions [112]. An allergy is a hypersensitivity reaction initiated by specific immune mechanisms. The clinical signs of food allergy may affect a variety of organs and systems, including the skin, gut, and respiratory, circulatory, and nervous systems. They can range from mild symptoms of hypersensitivity, such as itching in the mouth, to severe, systemic, and often fatal reactions, such as anaphylactic shock [113]. Food allergies are a common cause of skin disease in companion animals. Skin symptoms in dogs allergic to food may include itching, and erythematous dermatitis of the face, ear canals, armpits, groin, and paws. These symptoms are difficult to distinguish from atopic dermatitis (AD) and can be confused [64].

Research indicates that insects can also be a trigger for food allergies. An allergy related to the consumption of insects may be caused by a primary sensitization or a cross-reaction with another allergen [114].

In a study [64] conducted to investigate the interaction between *T. molitor* proteins and the immune system of dogs with clinical signs of allergy and allergy to storage mites compared to a group of clinically healthy dogs, no clear correlation was found between allergy to storage mites and the clinical condition of the dogs. In this study, the binding of canine serum IgE to mealworm larvae proteins was confirmed, but the differences between the healthy and allergic dogs were negligible. The results of these studies suggest that mite-allergic dogs may also clinically cross-react with mealworm proteins, so care should be taken in their case when using mealworm larvae as an alternative protein source in their diet.

Other hazards relate to the contamination of insects. For example, crickets can be contaminated with anthropogenic factors during breeding, packaging, cooking, or feeding [20]. *Staphylococcus aureus* can be used as an indicator of inadequate hygiene treatment. The research of Grabowski and Klein et al. [115] identified bacteria of the genus *Staphylococcus* spp. in crickets that were purchased at a pet store. Among the tested samples, *S. aureus* was identified in the species *Gryllus bimaculatus* (field cricket) [115]. Worryingly, insects may be vectors for antimicrobial resistance (AMR) genes [116,117,118,119].

It is significant that some fungi (including *Aspergillus* spp.) that are present in the intestines of crickets are capable of producing or modifying toxins. An extremely important issue that requires further research is the presence of mycotoxins in insects [20]. It was observed many years ago that *Aspergillus flavus* spores are highly pathogenic to freshly hatched *Musca domestica* by ingestion or even by contact [120]. It has been shown that the growth rate of yellow mealworm larvae is reduced by toxin T-2, aflatoxin B1, ochratoxin A, and rubratoxin B [121,122].

Research [123] showed the possibility of a bioaccumulation of heavy metals in insects. In these studies, *Gryllus assimilis* were exposed to diets enriched with zinc or cadmium. In the case of zinc, accumulation occurred only at the highest level of exposure, while for cadmium, even the lowest level of exposure of crickets to this element caused an increase in the concentration. It has been shown that cadmium concentrations are significantly higher than those allowed by Regulation (EU) 1881/2006 [124] and Regulation (EU) 1275/2013 [125].

In the case of BSFL, it has been shown that the absorption of heavy metals can influence their growth, but the effect of heavy metals on the BSFL gut microbiota is largely unknown. The studies by Wu et al. [126] analyzed the effect of Cu and Cd on the growth and intestinal microbiota of BSFL as well as the distribution of accumulated heavy metals in the larvae and their feces. It was shown that exposure to Cu and Cd did not significantly inhibit BSFL weight gain. At increased exposure doses, the contents of both Cu and Cd accumulated in BSFL bodies and feces increased significantly. The significant finding is that exposure to Cu and Cd significantly altered the intestinal BSFL microflora. Moreover, the bacterial diversity in the BSFL gut was significantly reduced after high metal exposure [126]. The studies of Proc et al. [127] demonstrated the ability of *H. illucens* to bioaccumulate selected elements. The accumulation of Cu, Fe, Hg, Mg, Mo, Se, and Zn occurred at all stages of the development of insects and in puparia. From a feed production point of view, it is obvious that the content of toxic heavy metals should be monitored. This phenomenon can have a negative effect not only on the larvae but also on consumers, as there is a risk of consuming the larvae containing heavy metals. Studies have been carried out [128] to determine whether the feed that feeds BSFL and the method of killing (blanching or freezing) will affect the bacterial load and heavy metal accumulation, ultimately affecting the safety of food containing BSFL. It was found that the method of killing had a significant effect on the microbial load and heavy metal content of the BSFL; as expected, blanching significantly reduced the microbial contamination of BSFL as well as resulting in slightly lower concentrations of heavy metals in the larvae. 

## 7. Conclusions

Insects can be used in the pet food industry. This is supported by the evolutionary adaptation of their wild ancestors to the collection of insects in the natural environment. The chemical composition of insects also corresponds to the nutritional requirements of this group of animals. They are characterized by a very good nutritional value (e.g., high protein content and high content of essential amino acids and fatty acids, including lauric acid), and products with them receive positive results in palatability tests. However, diets containing insect protein and their effects on animals require careful analysis, especially in terms of the risk of adverse food reactions, including allergic reactions that may be caused by insect consumption. Other hazards relate to the contamination of insects. They can be contaminated with anthropogenic factors during breeding, packaging, cooking, or feeding. These contaminants include the presence of bacteria, mold fungi, mycotoxins, and heavy metals, among others. 

## Figures and Tables

**Table 1:** Selected species of canines and the percentage of insects in their diets in the natural environment.

Species	Percentage of Diet/Chimney Content	Genus of Insects
*Atelocynus microtis* (Sclater, 1883) [30]	17% of intestine content	Coleoptera
*Canis lupus* (Linnaeus, 1758) [31]	1.2% of manure biomass	-
*Otocyon megalotis* (Desmarest, 1822) [32]	61.2% of diet	Isoptera, Coleoptera, Orthoptera
*Vulpes zerda* (Zimmermann, 1780) [33]	5.5% of manure biomass	Coleoptera, Hymenoptera, Isoptera
*Lycalopex culpaeus* (Molina, 1782) [30]	20% of diet	Coleoptera, Hymenoptera, Orthoptera

**Table 2:** The content of nutrients (% of dry matter—DM) and energy value (MJ) in selected forms of insects.

Species	Life Stage	Crude Protein	Crude Fat	Crude Fiber	Crude Ash	Gross Energy
Black soldier fly [18]	Larvae	42.35	24.90	7.00	21.50	22.10
Mealworm [39]	Larvae	53.75	37.10	-	2.75	26.85
	Imago	65.30	14.88	20.20	3.30	1.60
Banded cricket [40]	Imago	70.00	18.23	3.65	4.74	1.90
House cricket [41]	Nymph	67.25	14.41	15.72	4.80	17.32
	Imago	67.57	20.68	-	4.33	19.10
Field cricket [42]	Imago	56.40	28.80	7.00	6.40	21.50

**Table 3:** Essential amino acid (g/100 g of protein) contents in insect species and pork, beef, chicken, soybean meal, and fishmeal.

Item	Mealworm Larvae [47]	Black Soldier Fly Larvae [48]	House Cricket Imago [48]	Pork (*longissimus dorsi muscle*) [49]	Beef (Chuck) [50]	Chicken (Breast) [51]	Soybean Meal [52]	Fish Meal (*Peruvian anchovy*) [53]
Protein (g/100 g DM)	52.23	45.2	67.4	19.09	68.00	21.3	45.97	68.77
Arg	3.61	4.78	6.19	2.72	7.04	8.83	5.67	5.21
Val	3.62	9.03	9.36	3.67	6.59	4.79	4.33	5.38
Leu	4.22	7.23	8.00	4.27	9.09	7.09	8.04	7.66
Ile	2.51	4.73	4.91	2.39	5.65	3.90	4.00	4.00
Phe	2.51	4.38	3.77	3.1	4.54	3.71	5.99	3.36
Phe + tyr	6.62	10.58	11.77	4.82	8.53	6.81	9.21	6.28
Met	1.15	1.53	1.68	2.61	3.49	4.98	1.11	3.14
Met + Cys	3.42	2.79	2.76	2.81	4.97	6.01	2.05	4.19
Lys	3.03	7.43	6.41	3.96	9.79	9.95	5.44	7.63
His	1.60	3.21	2.63	2.6	4.32	3.47	3.00	2.08
Trp	0.57	1.46	1.04	0.23	1.37	2.07	1.65	0.99
Thr	2.42	4.18	3.90	3.05	5.04	4.93	4.81	4.14
Sum EAA	31.62	55.42	59.96	38.15	62.40	57.84	48.20	47.56

**Table 5:** Mineral composition (mg/100 g) in selected insect species and common livestock species.

Item	Black Soldier Fly (Larvae) [73,75]	House Cricket (Adult) [44]	Mealworm (Larvae) [77,78]	Duck (Breast) [79,80]	Pork (Semimembranosus Muscle) [81]	Chicken (Muscle) [82]	Lamb (Lean Lamb) [83]	Beef (Intercostal Muscles) [83]
**Macroelements**								
Ca	2900	140.3	43.5	8.2	11.8	11.1	16.1	6.1
P	350	842.4	706	205.7	225	134.4	195	182
Ca:P	8:1	1:6	1:16	1:25	1:19	1:12	1:12	1:29
K	57	365.3	947.9	227.2	280	206.4	303	266
Mg	24.5	127.9	202.7	21.4	26.6	17.9	23.5	21.2
Na	100	95	364.5	101.4	59.8	78.3	68.3	39.8
**Microelements**								
Zn	61.4	18.4	10.4	1.2	2.7	1.1	4.2	5.2
Fe	200	8.2	6.7	3.3	1.4	1.4	1.8	2.6
Mn	2	4.1	0.5	0.4	0.02	0.01	10.7	11.9
Cu	0.1	4.6	1.3	0.2	0.3	0.06	0.1	0.1

## Data Availability

Not applicable.

## References

[1] Legendre T.S., Baker M.A. (2020). Legitimizing edible insects for human consumption: The impacts of trust, risk–benefit, and purchase activism. J. Hosp. Tour. Res..

[2] Tomiyama J.M., Takagi D., Kantar M.B. (2020). The effect of acute and chronic food shortage on human population equilibrium in a subsistence setting. Agric. Food Secur..

[3] Baker M.A., Shin J.T., Kim Y.W. (2016). An exploration and investigation of edible insect consumption: The impacts of image and description on risk perceptions and purchase intent. Psychol. Mark..

[4] Van Huis A., Van Itterbeeck J., Klunder H., Mertens E., Halloran A., Muir G., Vantomme P. (2013). Edible Insects: Future Prospects for Food and Feed Security.

[5] Van Dijk M., Morley T., Rau M.L., Saghai Y. (2021). A meta-analysis of projected global food demand and population at risk of hunger for the period 2010–2050. Nat. Food..

[6] Alexander P., Rounsevell M.D.A., Dislich C. (2015). Drivers for global agricultural land use change: The nexus of diet, population, yield and bioenergy. Glob. Environ. Chang..

[7] Steinfeld H., Gerber P., Wassenaar T. (2006). Livestock’s Long Shadow: Environmental Issues and Options.

[8] Guyomard H., Bouamra-Mechemache Z., Chatellier V., Delaby L., Détang-Dessendre C., Peyraud J.L., Réquillart V. (2021). Review: Why and how to regulate animal production and consumption: The case of the European Union. Animal.

[9] (2022). A European Green Deal. https://ec.europa.eu/info/strategy/priorities-2019-2024/european-green-deal_en.

[10] Summary Report of the EAT-Lancet Commission. Healthy Diets from Sustainable Food Systems. https://thelancet.com/commissions/EAT.

[11] Aleksandrowicz L., Green R., Joy E.J.M., Smith P., Haines A. (2016). The impacts of dietary change on greenhouse gas emissions, land use, water use, and health. PLoS ONE.

[12] Boerema A., Peeters S., Swolfs F., Vandevenne S., Jacobs J., Staes P. (2016). Meire soybean trade: Balancing environmental and socio-economic impacts of an intercontinental market. PLoS ONE.

[13] Frewer L.J., Van der Lans I.A., Fischer A.R.H., Reinders M.J., Menozzi D., Zhang X., Van den Berg I., Zimmermann K.L. (2013). Public perceptions of agri-food applications of genetic modification—A systematic review and meta-analysis. Trends Food Sci. Technol..

[14] Weinrich R., Busch G. (2021). Consumer knowledge about protein sources and consumers’ openness to feeding micro-algae and insects to pigs and poultry. Future Foods.

[15] Janocha A., Milczarek A., Pietrusiak D., Łaski K., Saleh M. (2022). Efficiency of soybean products in broiler chicken nutrition. Animals.

[16] Sapkota T.B., Khanam F., Mathivanan G.P., Vetter S., Hussain S.G., Pilat A.L., Shahrin S., Hossain M.K., Sarker N.R., Krupnik T.J. (2021). Quantifying opportunities for greenhouse gas emissions mitigation using big data from smallholder crop and livestock farmers across Bangladesh. Sci. Total Environ..

[17] Calvez J., Gaudichon C. (2021). Insects on the menu: Characterization of protein quality to evaluate potential as an alternative protein source for human consumption. Am. J. Clin. Nutr..

[18] Makkar H., Tran G., Heuzé V., Ankers P. (2014). State-of-the-art on use of insects as animal feed. Anim. Feed Sci. Technol..

[19] Horne C.R., Hirst A.G., Atkinson D. (2015). Temperature-size responses match latitudinal-size clines in arthropods, revealing critical differences between aquatic and terrestrial species. Ecol. Lett..

[20] Fernandez-Cassi X., Supeanu A., Jansson A., Boqvist S., Vagsholm I. (2019). Novel foods: A risk profile for the house cricket (*Acheta domesticus*). EFSA J..

[21] Halloran A., Muenke C., Vantomme P., Van Huis A. (2014). Insects in the human food chain: Global status and opportunities. Food Chain.

[22] Tubiello F.N.N., Salvatore M., Cóndor Golec R.D.D., Ferrara A., Rossi S., Biancalani R., Federici S., Jacobs H., Flammini A. (2014). Agriculture, Forestry and Other Land Use Emissions by Sources and Removals by Sinks.

[23] Marberg A., Van Kranenburg H., Korzilius H. (2017). The big bug: The legitimation of the edible insect sector in the Netherlands. Food Policy.

[24] Maes J., Jacobs S. (2017). Nature-based solutions for Europe’s sustainable development. Conserv. Lett..

[25] Van Huis A., Oonincx D. (2017). The environmental sustainability of insects as food and feed. Agron. Sustain. Dev..

[26] FCI The Fédération Cynologique Internationale. http://www.fci.be/en/.

[27] The European Pet Food Industry Federation (2020). Annual Report. The Europeran Pet Food Industry.

[28] Kępińska-Pacelik J., Biel W. (2021). Microbiological hazards in dry dog chews and feeds. Animals.

[29] Bosch G., Zhang S., Oonincx D.G., Hendriks W.H. (2014). Protein quality of insects as potential ingredients for dog and cat foods. J. Nutr. Sci..

[30] Poyarkov A.D., Ovsianikov N.G., Sillero C., Hoffmann M., Macdonald D. (2004). Status Survey and Conservation Action Plan Canids: Foxes, Wolves, Jackals and Dogs. Canids: Foxes, Wolves, Jackals and Dogs. Status Survey and Conservation Action Plan.

[31] Nowak S., Mysłajek R., Kłosińska A., Gabrys G. (2011). Diet and prey selection of wolves *Canis lupus* ecolonizing Western and Central Poland. Mamm. Biol..

[32] Kuntzsch V., Nel J.A.J. (1992). Diet of bat-eared foxes *Otocyon megalotis* in the Karoo. Koedoe.

[33] Brahmi K., El Amine K., Mostéfaoui O., Baziz B., Aulagnier S. (2012). First quantitative data on the diet of the fennec fox, *Vulpes zerda* (*Canidae, Carnivora*), in Algeria. Folia Zool..

[34] McCusker S., Buff P.R., Yu Z., Fascetti A.J. (2014). Amino acid content of selected plant, algae and insect species: A search for alternative protein sources for use in pet foods. J. Nutr. Sci..

[35] Janssen R.H., Vincken J.P., Van den Broek L.A., Fogliano V., Lakemond C.M. (2017). Nitrogen-to-protein conversion factors for three edible insects: *Tenebrio molitor, Alphitobius diaperinus*, and *Hermetia illucens*. J. Agric. Food Chem..

[36] Homska N., Kowalska J., Bogucka J., Ziółkowska E., Rawski M., Kierończyk B., Mazurkiewicz J. (2022). Dietary fish meal replacement with *Hermetia illucens* and *Tenebrio molitor* larval meals improves the growth performance and nutriphysiological status of ide (*Leuciscus idus*) juveniles. Animals.

[37] Mæhre H.K., Dalheim L., Edvinsen G.K., Elvevoll E.O., Jensen I.J. (2018). Protein determination-method matters. Foods.

[38] Basto A., Matos E., Valente L.M.P. (2020). Nutritional value of different insect larvae meals as protein sources for European sea bass (*Dicentrarchus labrax*) juveniles. Aquaculture.

[39] Rumpold B.A., Schluter O.K. (2013). Nutritional composition and safety aspects of edible insects. Mol. Nutr. Food Res..

[40] Ramos-Elorduy J., Pino Moreno J.M., Correa S.C. (1998). Edible insects of the state of Mexico and determination of their nutritive values. Zoologia.

[41] Zielińska E., Baraniak B., Karaś M., Rybczyńska K., Jakubczyk A. (2015). Selected species of edible insects as a source of nutrient composition. Food Res. Intern..

[42] Józefiak D., Józefiak A., Kierończyk B., Rawski M., Świątkiewicz S., Długosz J., Engberg R.M. (2016). Insects—A natural nutrient source for poultry—A review. Ann. Anim. Sci..

[43] Vaga M., Berggren A., Jansson A. (2020). Growth, survival and development of house crickets (*Acheta domesticus*) fed flowering plants. J. Insects Food Feed.

[44] Udomsil N., Imsoonthornruksa S., Gosalawit C., Ketudat-Cairns M. (2019). Nutritional values and functional properties of house cricket (*Acheta domesticus*) and field cricket (*Gryllus bimaculatus*). Food Sci. Technol. Res..

[45] Bawa M., Songsermpong S., Kaewtapee C., Chanput W. (2020). Effect of diet on the growth performance, feed conversion, and nutrient content of the house cricket. J. Insect Sci..

[46] Huang C., Feng W., Xiong J., Wang T., Wang W., Wang C., Yang F. (2019). Impact of drying method on the nutritional value of the edible insect protein from black soldier fly (*Hermetia illucens* L.) larvae: Amino acid composition, nutritional value evaluation, in vitro digestibility, and thermal properties. Eur. Food Res. Technol..

[47] Wu R.A., Ding Q., Yin L., Chi X., Sun N., He R., Li Z. (2020). Comparison of the nutritional value of mysore thorn borer (*Anoplophora chinensis*) and mealworm larva (*Tenebrio molitor*): Amino acid, fatty acid, and element profiles. Food Chem..

[48] Matin N., Utterback P., Parsons C.M. (2021). True metabolizable energy and amino acid digestibility in black soldier fly larvae meals, cricket meal, and mealworms using a precision-fed rooster assay. Poult. Sci. J..

[49] Gan M., Shen L., Chen L., Jiang D., Jiang Y., Li Q., Chen Y., Ge G., Liu Y., Xu X. (2020). Meat quality, amino acid, and fatty acid composition of liangshan pigs at different weights. Animals.

[50] Wu G., Cross H.R., Gehring K.B., Savell J.W., Arnold A.N., McNeill S.H. (2016). Composition of free and peptide-bound amino acids in beef chuck, loin, and round cuts. Anim. Sci. J..

[51] Dalle Zotte A., Ricci R., Cullere M., Serva L., Tenti S., Marchesini G. (2020). Effect of chicken genotype and white striping–wooden breast condition on breast meat proximate composition and amino acid profile. Poult. Sci. J..

[52] Imelda J., Bhatnagar D. (2008). Effect of solid-state fermentation on nutrient composition of selected feed ingredients. Indian J. Fish..

[53] Li P., Wu G. (2020). Composition of amino acids and related nitrogenous nutrients in feedstuffs for animal diets. Amino Acids.

[54] Jiang X., Tian J., Hao Z., Zhang W. (2008). Protein content and amino acid composition in grains of wheat-related species. Agric. Sci. China..

[55] Ghosh S., Lee S.M., Jung C., Meyer-Rochow V.B. (2017). Nutritional composition of five commercial edible insects in South Korea. J. Asia Pac. Entomol..

[56] Zheng L., Hou Y., Li W., Yang S., Li Q., Yu Z. (2012). Biodiesel production from rice straw and restaurant waste employing black soldier fly assisted by microbes. Energy.

[57] Feng W.L., Qian L., Wang W.G., Wang T.L., Deng Z.K., Yang F., Xiong J., Wang C.W. (2018). Exploring the potential of lipids from black soldier fly: New paradigm for biodiesel production (II)—Extraction kinetics and thermodynamic. Renew. Energy.

[58] Wang C.W., Qian L., Wang W.K., Wang T.L., Deng Z.K., Yang F., Xiong J., Feng W.L. (2017). Exploring the potential of lipids from black soldier fly: New paradigm for biodiesel production (I). Renew. Energy.

[59] Chia S.Y., Tanga C.M., Osuga I.M., Cheseto X., Ekesi S., Dicke M., Van Loon J.J. (2020). Nutritional composition of black soldier fly larvae feeding on agro-industrial by-products. Entomol. Exp. Appl..

[60] Kipkoech C., Kinyuru J.N., Imathiu S., Roos N. (2017). Use of house cricket to address food security in Kenya: Nutrient and chitin composition of farmed crickets as influenced by age. Afr. J. Agric. Res..

[61] Ewald N., Vidakovic A., Langeland M., Kiessling A., Sampels S., Lalander C. (2020). Fatty acid composition of black soldier fly larvae (*Hermetia illucens*)—Possibilities and limitations for modification through diet. Waste Manag..

[62] Skřivanová E., Marounek M., Benda V., Brezina P. (2006). Susceptibility of *Escherichia coli*, *Salmonella* sp. and *Clostridium perfringens* to organic acids and monolaurin. Vet. Med..

[63] Finke M.D. (2013). Complete nutrient content of four species of feeder insects. Zoo Biol..

[64] Kim Y.B., Kim D.H., Jeong S.B. (2020). Black soldier fly larvae oil as an alternative fat source in broiler nutrition. Poult. Sci..

[65] Jayanegara A., Gustanti R., Ridwan R., Widyastuti Y. (2020). Fatty acid profiles of some insect oils and their effects on in vitro bovine rumen fermentation and methanogenesis. Ital. J. Anim. Sci..

[66] Cheseto X., Baleba S.B.S., Tanga C.M., Kelemu S., Torto B. (2020). Chemistry and sensory characterization of a bakery product prepared with oils from african edible insects. Foods.

[67] Almeida J.C.D., Perassolo M.S., Camargo J.L., Bragagnolo N., Gross J.L. (2006). Fatty acid composition and cholesterol content of beef and chicken meat in Southern Brazil. Braz. J. Pharm. Sci..

[68] Seo K., Cho H.-W., Chun J., Jeon J., Kim C., Kim M., Park K., Kim K. (2021). Evaluation of fermented oat and black soldier fly larva as food ingredients in senior dog diets. Animals.

[69] Jajic I., Popovic A., Urosevic M., Krstović S., Petrović M., Guljaš D., Samardzic M. (2020). Fatty and amino acid profile of mealworm larvae (*Tenebrio molitor* L.). Biotechnol. Anim. Husb..

[70] Zaki E. (2018). Fatty acids profile and quality characteristics of broiler chicken meat fed different dietary oil sources with some additives. Int. J. Health Anim. Sci. Food Saf..

[71] Flowers S., Hamblen H., Leal-Gutiérrez J.D., Elzo M.A., Johnson D.D., Mateescu R.G. (2018). Fatty acid profile, mineral content and palatability of beef from a multibreed Angus-Brahman population. Anim. Sci. J..

[72] Finke M.D., Capineira L. (2008). Nutrient content of insects. Encyclopedia of Entomology.

[73] Spranghers T., Ottoboni M., Klootwijk C., Ovyn A., Deboosere S., De Meulenaer B., Michiels J., Eeckhout M., De Clercq P., De Smet S. (2017). Nutritional composition of black soldier fly (*Hermetia illucens*) prepupae reared on different organic waste substrates. J. Sci. Food Agric..

[74] Oonincx D.G.A.B., Finke M.D. (2020). Nutritional value of insects and ways to manipulate their composition. J. Insects Food Feed.

[75] Liu X., Chen X., Wang H., Yang Q., Rehman K., Li W. (2017). Dynamic changes of nutrient composition throughout the entire life cycle of black soldier fly. PLoS ONE.

[76] Mlcek J., Rop O., Borkovcova M., Bednarova M. (2014). A comprehensive look at the possibilities of edible insects as food in Europe—A review. Polish J. Food Nutr. Sci..

[77] Ravzanaadii N., Kim S.H., Choi W.H., Hong S.J., Kim N.J. (2012). Nutritional value of mealworm *Tenebrio molitor* as food source. Int. J. Ind. Entomol..

[78] Finke M.D. (2002). Complete nutrient composition of commercially raised invertebrates used as food for insectivores. Zoo Biol..

[79] Ionita L., Popescu-Micloșanu E., Roibu C., Custură I. (2011). Bibliographical study regarding the quails’ meat quality in comparison to the chicken and duck meat. Lucr. Ştiinţifice.

[80] Kim D.H., Kim K.W., Kim Y.H., Kim J.A., Kim J., Moon K.D. (2015). Nutritional composition of horsemeat compared to white meat (chicken and duck). Korean J. Food Preserv..

[81] Tomović V.M., Petrović L.S., Tomović M.S., Kevrešan Ž.S., Džinić N.R. (2011). Determination of mineral contents of semimembranosus muscle and liver from pure and crossbred pigs in Vojvodina (northern Serbia). Food Chem..

[82] Sharipova A., Khaziev D., Kanareikina S., Kanareikin V., Rebezov M., Kazanina M., Andreeva A., Okuskhanova E., Yessimbekov Z., Bykova O. (2018). The effects of a probiotic dietary supplementation on the amino acid and mineral composition of broilers meat. Annu. Res. Rev. Biol..

[83] Purchas R.W., Wilkinson B.H.P., Carruthers F., Jackson F. (2014). A comparison of the nutrient content of uncooked and cooked lean from New Zealand beef and lamb. J. Food Compost. Anal..

[84] Hahn T., Tafi E., Paul A., Salvia R., Falabella P., Zibek S. (2020). Current state of chitin purification and chitosan production from insects. J. Chem. Technol. Biotechnol..

[85] Henriques B.S., Garcia E.S., Azambuja P., Genta F.A. (2020). Determination of chitin content in insects: An alternate method based on calcofluor staining. Front. Physiol..

[86] Smets R., Verbinnen B., Van De Voorde I., Aerts G., Claes J., Van Der Borght M. (2020). Sequential extraction and characterisation of lipids, proteins, and chitin from black soldier fly (*Hermetia illucens*) larvae, prepupae, and pupae. Waste Biomass Valorization.

[87] Jayanegara A., Haryati R.P., Nafisah A., Suptijah P., Ridla M., Laconi E.B. (2020). Derivatization of chitin and chitosan from black soldier fly (*Hermetia illucens*) and their use as feed additives: An in vitro study. Adv. Anim. Vet. Sci..

[88] Kulma M., Kouřimská L., Plachý V., Božik M., Adámková A., Vrabec V. (2019). Effect of sex on the nutritional value of house cricket, *Acheta domestica* L.. Food Chem..

[89] Wu Q., Patočka J., Kuča K. (2018). Insect antimicrobial peptides, a mini review. Toxins.

[90] Borrelli L., Varriale L., Dipineto L., Pace A., Menna L.F., Fioretti A. (2021). Insect derived lauric acid as promising alternative strategy to antibiotics in the antimicrobial resistance scenario. Front. Microbiol..

[91] Finke M.D. (2007). Estimate of chitin in raw whole insects. Zoo Biol..

[92] Gasco L., Finke M., Van Huis A. (2018). Can diets containing insects promote animal health?. J. Insects Food Feed.

[93] Mouithys-Mickalad A., Schmitt E., Dalim M., Franck T., Tome N.M., Van Spankeren M., Serteyn D., Paul A. (2020). Black soldier fly (*Hermetia illucens*) larvae protein derivatives: Potential to promote animal health. Animals.

[94] Di Mattia C., Battista N., Sacchetti G., Serafini M. (2019). Antioxidant activities in vitro of water and liposoluble extracts obtained by different species of edible insects and invertebrates. Front. Nutr..

[95] Klinger C., Gedon N., Udraite L., Hiltenkamp K., Mueller R., Böhm T. (2018). Effekt eines Insektenprotein-basierten Futters auf die Symptomatik von futtermittelallergischen Hunden. Tierarztl. Prax. Ausg. K Kleintiere Heimtiere.

[96] Premrov Bajuk B., Zrimšek P., Kotnik T., Leonardi A., Križaj I., Jakovac Strajn B. (2021). Insect protein-based diet as potential risk of allergy in dogs. Animals.

[97] Beynen A.C. (2018). Insect-based petfood. Creat. Companion.

[98] Leriche I., Fournel S., Chala V. (2017). Assessment of the digestive tolerance in cats of a new diet based on insects as the protein source. J. Feline Med. Surg..

[99] Kröger S., Heide C., Zentek J. Influence of proteins from the black soldier fly (*Hermetia illucens*) on nutrient digestibility and faecal and immunological parameters in dogs. Proceedings of the 21st European Society of Veterinary and Comparative Nutrition Congress.

[100] Abd El-Wahab A., Meyer L., Kölln M., Chuppava B., Wilke V., Visscher C., Kamphues J. (2021). Insect larvae meal (*Hermetia illucens*) as a sustainable protein source of canine food and its impacts on nutrient digestibility and fecal quality. Animals.

[101] Penazzi L. (2021). In vivo and in vitro digestibility of an extruded complete dog food containing black soldier fly (*Hermetia illucens*) larvae meal as protein source. Front. Vet. Sci..

[102] Freel T.A., McComb A., Koutsos E.A. (2021). Digestibility and safety of dry black soldier fly larvae (BSFL) meal and BSFL oil in dogs. J. Anim. Sci..

[103] Areerat S., Chundang P., Lekcharoensuk C., Kovitvadhi A. (2021). Possibility of using house cricket (*Acheta domesticus*) or mulberry silkworm (*Bombyx mori*) pupae meal to replace poultry meal in canine diets based on health and nutrient digestibility. Animals.

[104] Bosch G., Swanson K. (2020). Effect of using insects as feed on animals: Pet dogs and cats. J. Insects Food Feed.

[105] Kierończyk B., Rawski M., Pawełczyk P., Różyńska J., Golusik J., Mikołajczak Z., Józefiak D. (2018). Do insects smell attractive to dogs? A comparison of dog reactions to insects and commercial feed aromas—A preliminary study. Ann. Anim. Sci..

[106] Kilburn L.R., Carlson A.T., Lewis E., Rossoni Serao M.C. (2020). Cricket (*Gryllodes sigillatus*) meal fed to healthy adult dogs does not affect general health and minimally impacts apparent total tract digestibility. J. Anim. Sci..

[107] Kowalska J., Rawski M., Homska N., Mikołajczak Z., Kierończyk B., Świątkiewicz S., Wachowiak R., Hetmańczyk K., Mazurkiewicz J. (2022). The first insight into full-fat superworm (*Zophobas morio*) meal in guppy (*Poecilia reticulata*) diets: A study on multiple-choice feeding preferences and growth performance. Ann. Anim. Sci..

[108] Rawski M., Mazurkiewicz J., Kierończyk B., Józefiak D. (2020). Black soldier fly full-fat larvae meal as an alternative to fish meal and fish oil in siberian sturgeon nutrition: The effects on physical properties of the feed, animal growth performance, and feed acceptance and utilization. Animals.

[109] Sungmun B., Seulbi L., Jongwon K., Yeonhyeon H. (2020). Analysis of consumer receptivity to pet food containing edible insects in South Korea. J. Appl. Entomol..

[110] Naranjo-Guevara N., Fanter M., Conconi A.M., Floto-Stammen S. (2021). Consumer acceptance among Dutch and German students of insects in feed and food. Food Sci. Nutr..

[111] Guiné R.P.F., Correia P., Coelho C., Costa C.A. (2021). The role of edible insects to mitigate challenges for sustainability. Open Agric..

[112] Broekman H.C.H.P., Knulst A.C., De Jong G., Gaspari M., Den Hartog Jager C.F., Houben G.F., Verhoeckx K.C.M. (2017). Is mealworm or shrimp allergy indicative for food allergy to insects?. Mol. Nutr. Food Res..

[113] De Martinis M., Sirufo M.M., Suppa M., Ginaldi L. (2020). New perspectives in food allergy. Int. J. Mol. Sci..

[114] de Gier S., Verhoeckx K. (2018). Insect (food) allergy and allergens. Mol. Immunol..

[115] Grabowski N.T., Klein G. (2017). Microbiology of cooked and dried edible Mediterranean field crickets (*Gryllus bimaculatus*) and superworms (*Zophobas atratus*) submitted to four different heating treatments. Food Sci. Technol. Int..

[116] Channaiah L.H., Subramanyam B., Mckinney L.J., Zurek L. (2010). Stored-product insects carry antibiotic-resistant and potentially virulent enterococci. FEMS Microbiol. Ecol..

[117] Hemmatinezhad B., Ommi D., Hafshejani T.T., Khamesipour F. (2015). Molecular detection and antimicrobial resistance of *Pseudomonas aeruginosa* from houseflies (*Musca domestica*) in Iran. J. Venom. Anim. Toxins Incl. Trop. Dis..

[118] Lowe C.F., Romney M.G. (2011). Bedbugs as vectors for drugresistant bacteria. Emerg. Infect. Dis..

[119] Tian B., Fadhil N.H., Powell J.E., Kwong W.K., Moran N.A. (2012). Long-term exposure to antibiotics has caused accumulation of resistance determinants in the gut microbiota of honeybees. mBio.

[120] Amonkar S.V., Nair K.K. (1965). Pathogenicity of Aspergillus flavus link to Musca domestica nebulo fabricius. J. Invertebr. Pathol..

[121] Davis G.R.F., Schiefer H.B. (1982). Effects of dietary T-2 toxin concentrations fed to larvae of the yellow mealworm at three dietary protein levels. Comp. Biochem. Physiol. Part C Comp. Pharmacol..

[122] Davis G.R.F. (1982). Growth of larvae of *Tenebrio molitor* L. fed diets containing penicillic acid, aflatoxin B, ochratoxin A, or rubratoxin B at three dietary protein levels. Arch. Int. Physiol. Biochim..

[123] Bednarska A.J., Opyd M., Żurawicz E., Laskowski R. (2015). Regulation of body metal concentrations: Toxicokinetics of cadmium and zinc in crickets. Ecotoxicol. Environ. Saf..

[124] European Commission (EC) (2006). Commission Regulation (EC) No.1881/2006 of 19 December 2006 on setting maximum levels for certain contaminants in foodstuffs. Off. J. Eur. Union L.

[125] European Commission (EC) (2013). Commission Regulation (EC) No. 1275/2013 of 6 December 2013 amending Annex I to Directive 2002/32/EC of the European Parliament and of the Council as regards maximum levels for arsenic, cadmium, lead, nitrites, volatile mustard oil and harmful botanical impurities. Off. J. Eur. Union L.

[126] Wu N., Wang X., Xu X., Cai R., Xie S. (2020). Effects of heavy metals on the bioaccumulation, excretion and gut microbiome of black soldier fly larvae (*Hermetia illucens*). Ecotoxicol. Environ. Saf..

[127] Proc K., Bulak P., Wiącek D., Bieganowski A. (2020). *Hermetia illucens* exhibits bioaccumulative potential for 15 different elements—Implications for feed and food production. Sci. Total Environ..

[128] Bessa L.W., Pieterse E., Marais J., Dhanani K., Hoffman L.C. (2021). Food safety of consuming black soldier fly (*Hermetia illucens*) larvae: Microbial, heavy metal and cross-reactive allergen risks. Foods.

